# Enhanced Defect Detection in Carbon Fiber Reinforced Polymer Composites via Generative Kernel Principal Component Thermography

**DOI:** 10.3390/polym13050825

**Published:** 2021-03-08

**Authors:** Kaixin Liu, Zhengyang Ma, Yi Liu, Jianguo Yang, Yuan Yao

**Affiliations:** 1 Institute of Process Equipment and Control Engineering, Zhejiang University of Technology, Hangzhou 310023, China; kxliu@zjut.edu.cn (K.L.); zymazjut@163.com (Z.M.); yangjg@zjut.edu.cn (J.Y.); 2 Department of Chemical Engineering, National Tsing Hua University, Hsinchu 30013, Taiwan

**Keywords:** infrared non-destructive assessment, polymer composite, deep learning, generative adversarial network, thermographic data analysis, kernel principal component analysis

## Abstract

Increasing machine learning methods are being applied to infrared non-destructive assessment for internal defects assessment of composite materials. However, most of them extract only linear features, which is not in accord with the nonlinear characteristics of infrared data. Moreover, limited infrared images tend to restrict the data analysis capabilities of machine learning methods. In this work, a novel generative kernel principal component thermography (GKPCT) method is proposed for defect detection of carbon fiber reinforced polymer (CFRP) composites. Specifically, the spectral normalization generative adversarial network is proposed to augment the thermograms for model construction. Sequentially, the KPCT method is used by feature mapping of all thermogram data using kernel principal component analysis, which allows for differentiation of defects and background in the dimensionality-reduced data. Additionally, a defect-background separation metric is designed to help the performance evaluation of data analysis methods. Experimental results on CFRP demonstrate the feasibility and advantages of the proposed GKPCT method.

## 1. Introduction

Infrared thermography (IRT), an attractive non-destructive assessment technique, is applied widely in the quality assessment of composite structures due to its fast response, wide-range of measurement, and intuitive results [1,2,3,4]. However, especially in the task of defect assessment, IRT is difficult to achieve the desired inspection results. The non-uniform background and noise in thermal images often hinder the accuracy and efficiency of IRT detection due to the influence of experimental setup and environment [5,6]. Therefore, it has become common to adopt thermographic data analysis methods to improve the IRT performance.

In recent years, machine learning methods have provided alternative solutions and achieved promising results for industrial data/image analysis tasks [7,8,9]. For IRT defect identification, several thermographic data analysis methods have been developed to enhance the visibility of defects in thermograms. These methods include principal component thermography (PCT) [10] and its variants (such as sparse PCT [11], candid covariance-free incremental PCT [12], generative PCT [13]), thermographic signal reconstruction [14], penalized least squares [15], pulsed phase thermography [16], independent component thermography [17], manifold learning methods [18,19,20], autoencoder methods [21,22], other deep learning methods [23,24,25], etc. Most of the above-mentioned methods can be considered as a kind of metric learning [26]. For example, PCT is an unsupervised linear metric learning method with simplicity and scalability, which can be used to improve the defect detection performance of IRT.

However, the thermographic data recorded by IRT are often nonlinear due to uneven heating and thermal anisotropy of composite materials [21]. Direct observation of this property is that thermal images contain heavily inhomogeneous background and noise, which masks defect information. Therefore, nonlinear models may be more suitable than linear candidates in the task of defect identification. Nevertheless, there are only a few nonlinear thermographic data analysis methods developed [18,19,20]. Another challenge of thermographic data analysis is that the thermograms are typically limited because of the shallow detection depth of the thermal imager, which indeed restricts the defect detection capabilities of thermal imaging methods. While this difficulty is solved by using a data expansion strategy in the generative PCT (GPCT) approach [13], there is still room for improvement in the performance of defect identification because the nonlinearity of thermal image data is not considered in it.

In this work, a generative kernel principal component thermography (GKPCT) method for defect detection of carbon fiber reinforced polymers is proposed to enhance the visibility of defects. The main contributions are summarized as the following three aspects. First, a kernel principal component thermography (KPCT) method is developed to extract nonlinear features from thermographic data. Second, a spectral normalized generative adversarial network (SNGAN) [27] is integrated with KPCT to build a GKPCT modeling and analysis framework. A data augmentation strategy is proposed to generate a number of informative thermographic images to improve the feature mapping performance of the KPCT model, thus improving the defect detection. Third, a local pixel standard deviation (LPSD) evaluation index is proposed for comparing different thermal image analysis methods for defect detection. Two case studies demonstrate the feasibility and advantages of the GKPCT approach.

The rest parts are so organized. In Section 2, the framework of GKPCT with its algorithmic details is proposed. Section 3 introduces the experimental research of IRT defect detection on two carbon fiber reinforced polymer (CFRP) specimens. In Section 4, experimental results are reported. Finally, the conclusions are summarized in Section 5.

## 2. Methodology

### 2.1. KPCT–Nonlinear Thermographic Data Analysis Approach

Since thermographic data tend to be nonlinear, a linear thermography approach based on dimensionality reduction can lose the high-dimensional structure of the data. Therefore, a nonlinear model may be applicable and worth trying in the analysis of thermographic data. In this work, the KPCT method is proposed for learning nonlinear features in thermograms, so that linearly indistinguishable defects are separated from the background. Its principle can be summarized as mapping the original thermographic data into a high-dimensional feature space H by a nonlinear transformation Φ, followed by principal component analysis (PCA) in H [28]. As a result, KPCT solves the problem of nonlinearity and feature extraction of thermal image data, and the defect morphology is identified. As a point of attraction for this work, it is worth illustrating that nonlinear mappings are learned through kernel methods, which is different from the developed methods for manifold and deep learning thermographic data analysis [18,19,20,21,22,23,24,25]. The following details can help to understand the technical process of the KPCT method.

The KPCT approach begins with IRT experiments. After the experiment, an infrared dataset is obtained, which can be expressed as a three-dimensional (3–D) matrix with dimensions of *n_t_ × n_x_* × *n_y_*. It means that *n_t_* frame thermal images with a size of *n_x_* × *n_y_* are selected as the original data for modeling. To meet the needs of KPCT data analysis later, this 3-D data is converted into a two-dimensional (2-D) matrix **X** with *n_t_* × (*n_x_* × *n_y_*) dimensions. Subsequently, a two-step data preprocessing procedure is executed on the **X**. In detail, first, the rows of the matrix are subjected to a max-min normalization to unify the scale to 0–1, after which the columns are centered to remove the inhomogeneous background of the data.

After the data is prepared, KPCT selects pixel temperature signals (column vectors $x_{i}$ of matrix **X**) as samples and projects them to high-dimensional feature space H through nonlinear mapping Φ. The covariance matrix in the H is:

(1) $$S^{H}=\frac{1}{m}\sum_{i=1}^{m}\Phi (x_{i})\Phi {\left(x_{i}\right)}^{T},$$

where $\Phi (x_{i})$ is a sample in feature space H, *m* is the number of samples and *m* = *n_x_* × *n_y_*.

Because $\Phi (x_{i})$ is unknown, the eigenvalues and eigenvectors of $S^{H}$ cannot be obtained directly through PCA. Herein, defining an *m* × *m* kernel matrix $K=\Phi {\left(x_{i}\right)}^{T}\Phi (x_{i})$, and the eigenvalue decomposition of **K** can be expressed as:

(2) Kw=λw,

where λ is the eigenvalue matrix of **K**, **w** is the corresponding eigenvector matrix.

Equation (2) is multiplied by $\frac{1}{m}\sum_{i=1}^{m}\Phi (x_{i})$ on the left to perform identity transformation to obtain:

(3) $$\frac{1}{m}\sum_{i=1}^{m}\Phi (x_{i})[\Phi {\left(x_{i}\right)}^{T}\Phi (x_{i})w]=\frac{1}{m}\sum_{i=1}^{m}\Phi (x_{i})\lambda w.$$

The combined Equations (1) and (3) are equivalent to the following equation:

(4) $$S^{H}[\Phi (x)w]=\lambda [\Phi (x)w].$$

Observing the above equation shows that the loading matrix of $S^{H}$ is exactly Φ(x)w. Although Φ(x) is unknown, the loading matrix is not the ultimate goal. The component of the sample Φ(x) in the projection direction Φ(x)w is $\Phi {\left(u\right)}^{T}\Phi (u)w$. Consequently, the score matrix **T** of the KPCT of can be calculated as:

**T** = **Kw**. (5)

At this point, the key to solving the matrix **T** is to calculate the **K** matrix. After that, the **w** matrix is calculated by the formula (2). In this work, the **K** matrix is calculated by the Gaussian kernel function, and its mathematical expression is as follows:

(6) $$K_{ij}=\exp(-\frac{{\left\|x_{i}-x_{j}\right\|}^{2}}{\tau }),$$

where **x***_i_*, **x***_j_* for the *i*-th and *j*-th samples of the original data, respectively, and τ is the width of the Gaussian kernel.

After the $T=R^{m\times d}$ matrix is obtained, an image reconstruction procedure is applied to visualize the defects. Specifically, the column vector of the matrix **T** is reconstructed as a 2–D matrix of size *n_x_* × *n_y_*, and *d* principal components (PCs) thermal images are obtained accordingly. However, checking each PC image is both time-consuming and laborious. As a variant of the PCT method, as with PCT, a few PCs retain most of the information about the original thermographic data [8]. Therefore, only the first few columns of **T** need to be investigated to reconstruct the PC images. These PC images contain more pronounced geometric properties of defects than the original thermograms, which shows the defect information (including position, shape, and size) more clearly than the original thermal images.

### 2.2. GKPCT-KPCT Approach Based on Image Augmentation

Recently, the GAN family has become increasingly popular and has demonstrated attractive performance in data augmentation tasks [29,30]. SNGAN, an improved GAN method using weight normalization (also known as spectrum normalization) technique was proposed to address the problem of unstable GAN training [27]. Like GAN, SNGAN has basically the same structure: a generator *G* for learning the model distribution and a discriminator *D* for distinguishing the model distribution from the target. Different from GAN, in SNGAN’s discriminator network, the weight matrix of each layer is spectrally normalized. The advantage of this is that SNGAN is stable and easy to implement during training [27]. The details of the algorithm can be described as follows:

The weight matrix $W^{l}$ is spectrally normalized for each layer of *D* so that it satisfies the Lipschitz constraint $\sigma (W^{l})=1$:

(7) $${\bar{W}}_{\mathrm{SN}}=\frac{W^{l}}{\sigma (W^{l})}.$$

where $W^{l}$ represents the weight matrix of layer *l* (*l* = 1, …, *L*), $\sigma (W^{l})$ denotes the spectral norm of $W^{l}$, ${\bar{W}}_{\mathrm{SN}}$ is the weight matrix after spectral normalization.

**Figure 1:**
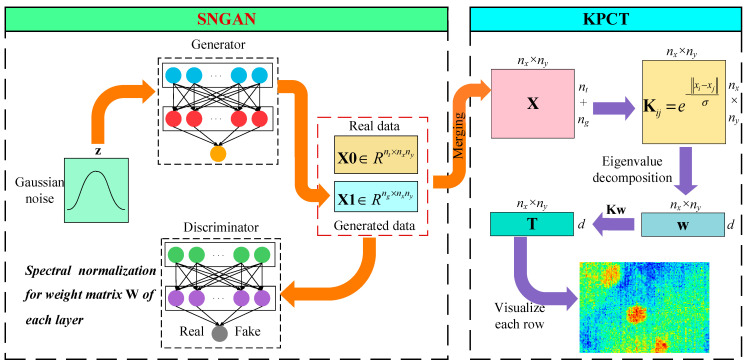
The GKPCT modeling and analysis framework.

Here, SNGAN is introduced as an image augmentation strategy into the IRT data analysis model to expand the number of thermal images, and a SNGAN-based GKPCT is proposed to improve the defect detection performance of KPCT. The motivation for this is that thermal image analysis models often perform poorly due to insufficient training data. As shown in Figure 1, the entire GKPCT framework consists of two parts: the SNGAN thermal image generation structure on the left and the KPCT thermal image analysis structure on the right. With the initial infrared dataset, SNGAN architecture is implemented to generate thermal image sequences. The convolutional layers of the two networks both use the rectified linear unit (ReLU) activation function to prevent the gradient from over-expanding. The biggest difference between them is that each layer weight matrix of the D network is spectrally normalized. In addition, all convolution kernel dimensions are set to 3 × 3, the optimizer selected is Adam, and the best learning rate is set to 0.0002.

After the above SNGAN model was trained, *n_g_*-frame fake images with a similar distribution to the original thermal image data were generated and expanded to the training data. A merged thermographic data with a size of (*n_t_* + *n_g_*) × (*n_x_* × *n_y_*) is used for subsequent KPCT analysis. Consequently, GKPCT eliminates most of the inhomogeneous background and makes the geometric characteristics of the defect areas visible in the PC images, thus improving the efficiency and accuracy of defect assessment by IRT techniques.

## 3. Experiments

### 3.1. Manufacturing and Data Acquisition of CFRP Specimen 1

To test the effectiveness of the proposed GKPCT, IRT defect detection and data analysis were performed on the CFRP specimen. Considering that the defect properties of the natural CFRP are unknown, it is difficult for GKPCT to judge the performance of its defect assessment. In this experiment, an artificial CFRP specimen containing multiple defects was manufactured. It is made from 20 carbon fiber sheets and epoxy resin using the resin transfer modeling [31] process, with an overall thickness of about 1 cm and dimensions of about 18 cm × 18 cm. In more detail, the CFRP was fabricated by injecting thermosetting resin into fiber preformed rods placed in a closed mold. Prior to resin injection, three Teflon strips (of different shapes but all equal to about 3 cm^2^ in area) were inserted into the carbon fiber sheet to form the preset defects.

**Figure 2:**
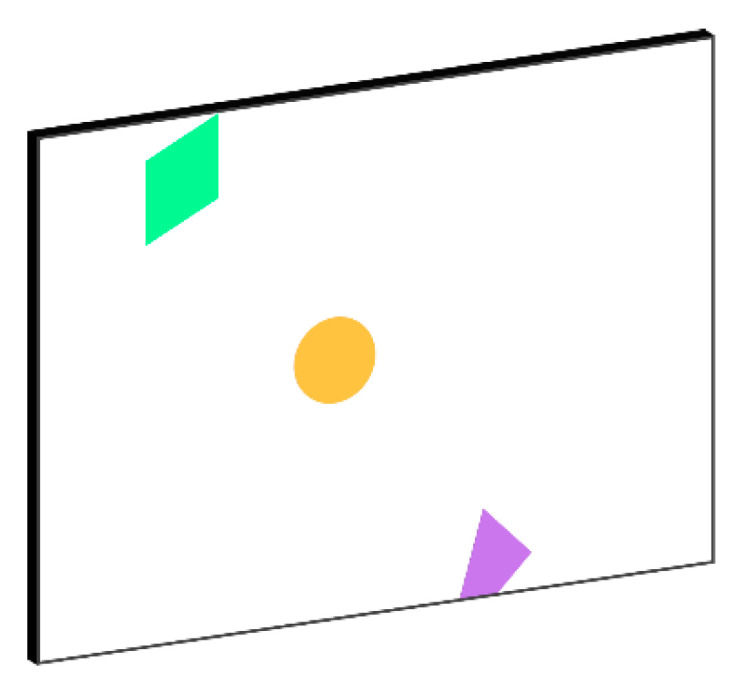
Illustration of defective regions in a CFRP specimen 1.

As shown in Figure 2, the defect pattern inside the artificially fabricated CFRP specimen 1 can be observed. From the top view, the deepest diamond-shaped defect is in the upper left corner where the Teflon strip is covered by three layers of carbon fiber sheets (about 0.15 cm deep). The shallowest Teflon strip is covered by a layer of carbon fiber sheet (about 0.05 cm deep), which is a trapezoidal defect located in the lower right corner. The one remaining defect is circular (about 0.10 cm deep) in the middle position made of a Teflon strip covered by two layers of carbon fiber sheets. It needs to be noted that these defects are invisible after the epoxy is injected. The purpose of this work is to develop a thermography data analysis method that can facilitate IRT defect detection performance, leading to more accurate defect estimation.

**Figure 3:**
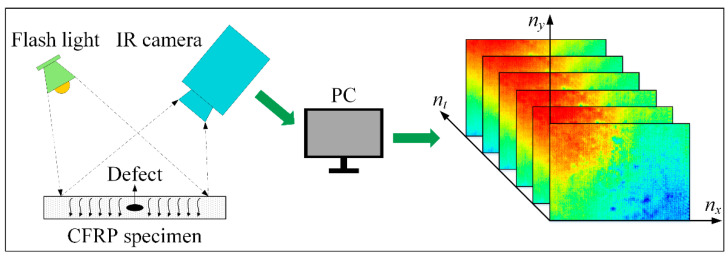
The PT inspection equipment and acquired thermogram sequence.

Pulsed thermography (PT), one of the fastest and simplest IRT techniques, was used in this study [16,17]. The experimental setup of the PT system in our laboratory is shown in Figure 3, which consists of a flashlight that can deliver 3200 W of energy for 3 ms duration, an infrared camera (TAS-G100EXD, NEC, Minato-ku, Tokyo, Japan.) with a COMS sensor and an uncooled focal plane array (UFPA) detector, and a computer for image storage and analysis. Specifically, the process of PT detecting CFRP internal defects is:

The CFRP specimen 1 is heated by a thermal pulse excited by a working flash, which passes through the specimen after the thermal front contacts the surface. Typically, surface temperatures in healthy areas will decrease evenly over time. However, subsurface discontinuities caused by defects can cause the surface to produce an abnormal temperature response. This temperature pattern can be recorded during the specimen 1 cooling phase by the infrared camera. The camera is sensitive to infrared light in the temperature range of −40 to 1500 °C with a spectral range of 8–14 µm. Its sampling frequency and spatial resolution of the camera are 30 frames per second and 320 × 240 pixels, respectively. At the end of the experiment, 308 × 212 pixels were selected as the region of interest (ROI) for each thermogram. As a result, 90 frames of thermal images were captured within 3 s for subsequent analysis.

**Figure 4:**
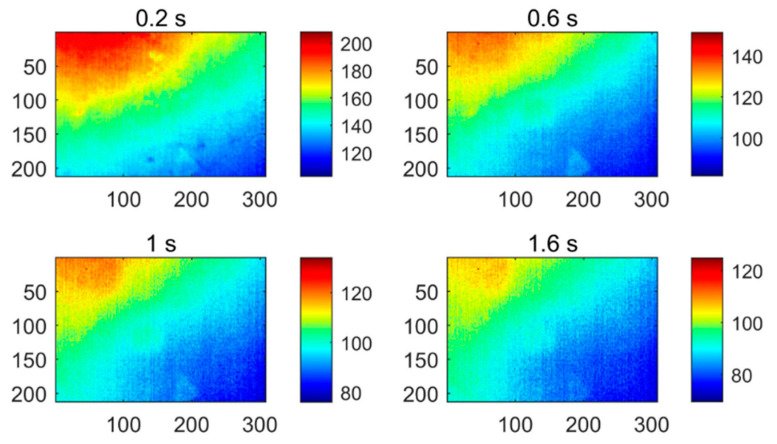
The PT thermograms of specimen 1 recorded at different sampling instances.

Figure 4 shows four of the thermograms obtained by implementing the PT technique for testing CFRP specimen 1, which were sampled at different instances. It can be found that the thermograms contain a large amount of uneven background and noise, which masks the defect information. This result can be attributed to the limited detection capability of the PT system itself. PT is easily affected by various factors, such as reflections from the environment, changes in emissivity, uneven heating, and changes in the surface geometry of the detection target. Although the effects of similar surface conditions can be controlled, energy inhomogeneity is an inevitable problem in the experimental environment. It is for this reason that more and more thermal image analysis methods are developed to promote the performance of PT.

### 3.2. Manufacturing and Data Acquisition of CFRP Specimen 2

**Figure 5:**
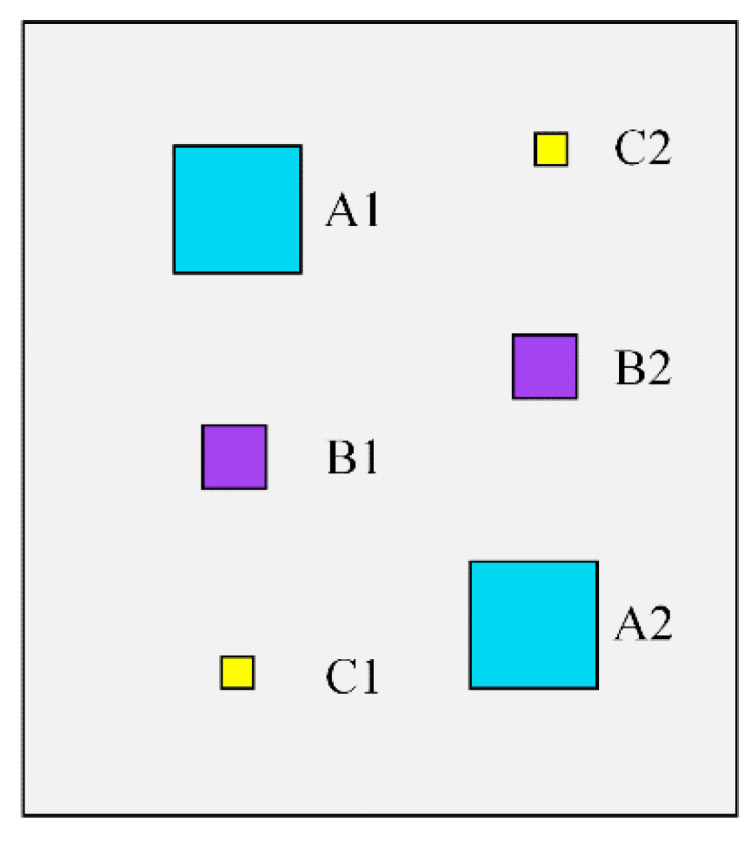
Illustration of defective regions in a CFRP specimen 2.

**Figure 6:**
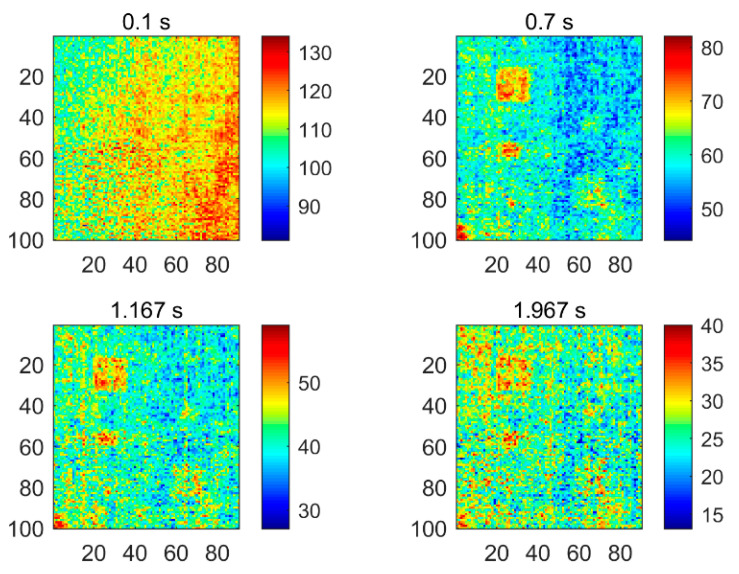
The PT thermograms of specimen 2 recorded at different sampling instances.

The second CFRP specimen was manufactured in a similar way to the first one, which contains six defects. Figure 5 shows the defect distribution in the region of interest of this specimen. The six defects are square and range in size from 1.6 cm × 1.6 cm to 0.4 cm × 0.4 cm. The three defects on the left, i.e., defects A1, B1, and C1, are located under one layer of carbon fiber sheet, while the defects on the right, i.e., defects A2, B2, and C2, are located under two layers of sheets. After the specimen was fabricated, the PT technique was used for nondestructive testing. A total of 59 thermal images with a size of 90 × 100 were obtained in the experiment for the subsequent analysis. Figure 6 shows some of the original thermal images recorded at different moments, from which it can be observed that the defects are difficult to be identified due to the existence of inhomogeneous background and noise.

## 4. Results and Discussion

To evaluate the experimental results more objectively, a commonly-used signal-to-noise ratio (*SNR*) index is introduced [10,17,32,33,34]. Its mathematical expression is as follows:

(8) $$SNR=\frac{M_{def}-M_{in}}{\sigma_{in}},$$

where $M_{def}$ is the mean of pixel values in the defective area, $M_{in}$ is the mean of pixel values in the non-defective area, and $\sigma_{in}$ is the standard deviation of pixel values in the non-defective area. The higher the SNR value, the greater the ability of the method to identify defects. Therefore, the SNR index allows comparing the performance of different thermographic data analysis methods.

### 4.1. Thermal Image Data Analysis of Specimen 1

In this section, PCT, GPCT are used to compare with the proposed KPCT and GKPCT methods. PCT, which uses PCA techniques to process and analyze thermal images [12]. As a representative data compression method, PCT has the advantages of high noise reduction capabilities, no parameter limitations, and low computational cost in the field of thermography. And GPCT is a deep learning thermographic data analysis method developed on the basis of PCT to address the shortage of training data. The results of PCT and GPCT on CFRP specimens with three defects are shown in Figure 5 and Figure 6, respectively.

**Figure 7:**
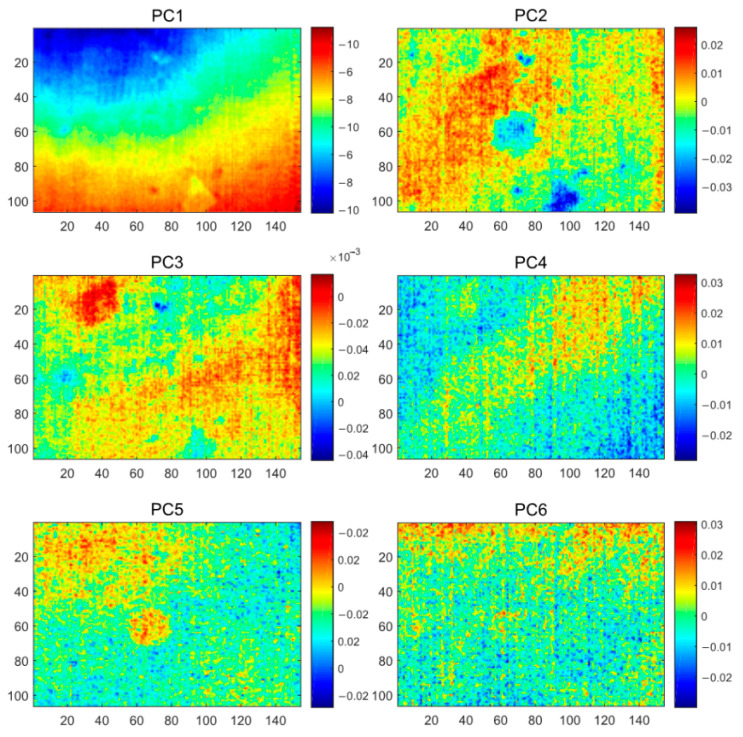
The PCT analysis results of the CFRP specimen 1.

The PCT results, as shown in Figure 7, the first six PC plots show three types of information as inhomogeneous background, noise, and defects. PC1 shows a heavily inhomogeneous background, while PC2 and PC3 highlight the geometric properties of the defects at different depths. Compared to the original PT images, the PCT method better reveals the number and rough location of the defects. However, as a linear analysis method, PCT neither completely separates the background and defects in the thermographic data, nor does it incorporate all the defects that are centralized in a single PC for easy manual inspection.

**Figure 8:**
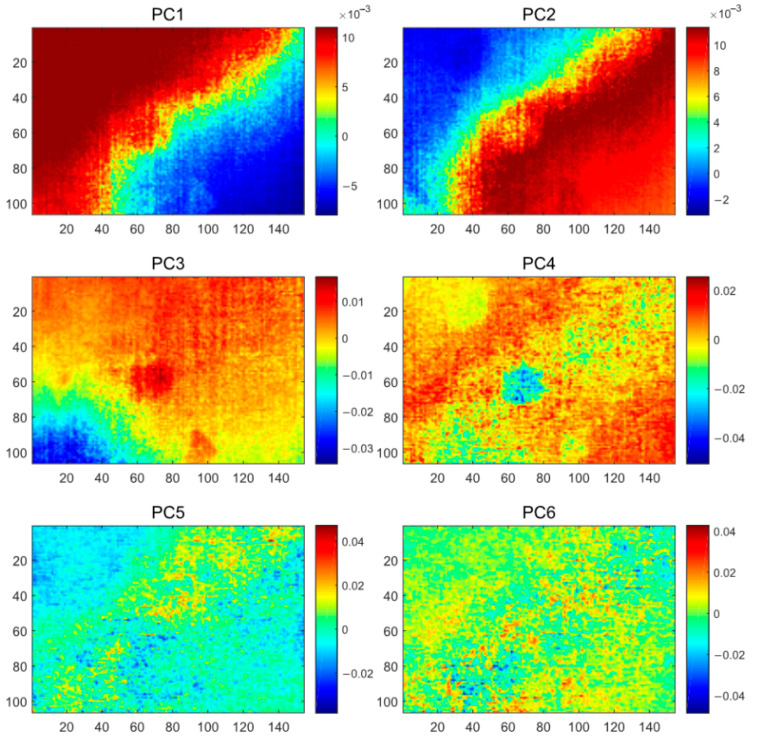
The GPCT analysis results of the CFRP specimen 1.

As with PCT, the first six PCs of GPCT were visualized as in Figure 8. Even though GPCT overcame the challenge of insufficient training data for the PCT method, the expected improvement in defect detection was not achieved. PCs 1–4 contained severe inhomogeneous backgrounds, which added to the difficulty of defect identification. Compared with PCT, the defect detection ability of GPCT becomes worse, which may be attributed to the poor nonlinear processing ability of the GPCT method that extracts the inhomogeneous background as the main information after the data augmentation, and the defect information is not focused on. However, although the defect information is masked by the inhomogeneous background, the boundaries of defects in the GPCT results (PC3 and PC4) are still clearer than those in the PCT results (PC2) under careful observation.

**Figure 9:**
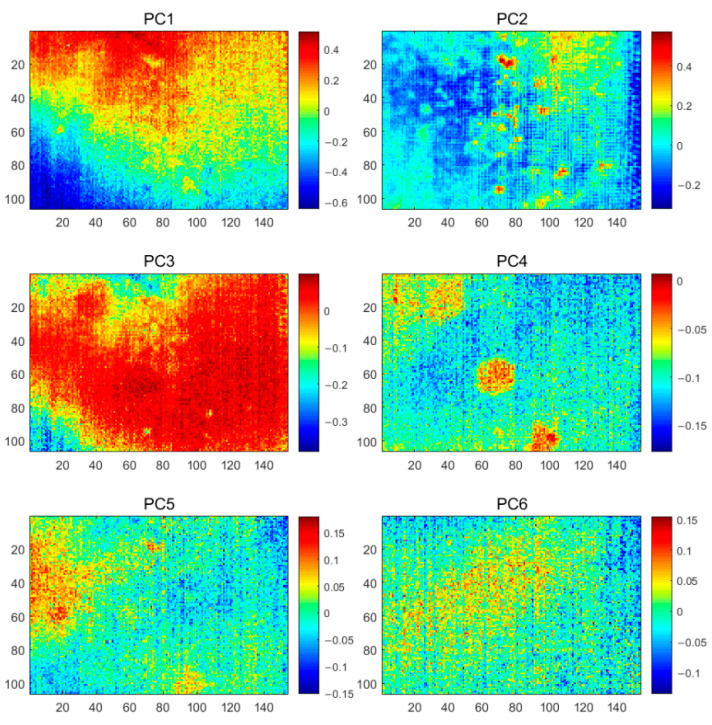
The KPCT analysis results of the CFRP specimen 1.

KPCT, application of the kernel PCA algorithm to thermography. The value of the Gaussian kernel width was chosen to be 1.4 in the execution of the algorithm. By mapping the nonlinearly indistinguishable thermographic data into a high-dimensional space, it can be linearly separable. In doing so, the performance of KPCT is usually improved over PCT (besides the identification of diamond-shaped defects), which has a stronger ability to extract defect information. Figure 9 shows the first six PC images obtained by KPCT. It can be observed from the figure that the PC4 image shows all the prefabricated defects, while PC1 and PC3 focus on the severe uneven background. Compared to PCT, the defects were concentrated in a single PC4 thermal image in the results of KPCT. However, the KPCT still detects defects with poor visibility and the profile of the defects is not clear. As shown in the PC3 image, the faintly visible defect is covered by a severely uneven background. One of the main reasons for the unsatisfactory performance of KPCT is that the proportion of information of uneven background and defect features is unbalanced in the original thermal image. The nonlinear mapping capability of KPCT is used more to extract the inhomogeneous background. Comparing Figure 7 and Figure 9, the first six PCs of KPCT contribute more inhomogeneous background information (KPCT has two PC images, i.e., PCI and PC3, whereas PCT has only one PC1 image).

**Figure 10:**
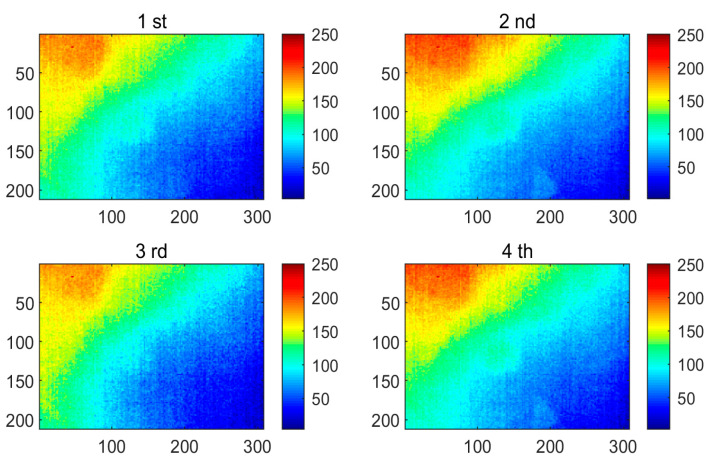
Several simulated SNGAN thermograms of the CFRP specimen 1.

In this work, the GKPCT method is applied to analyze the thermograms to enhance the visibility of defects. The algorithm is calculated and implemented based on the TensorFlow framework on a computer with 16 G memory, Intel^®^ Core™ i7 CPU, and Windows 7 system. The value of the Gaussian kernel width τ was chosen to be 5.6 in the algorithm. As the core of the GKPCT approach, there is a need to determine how many thermal images generated by SNGAN are beneficial for overall model performance. To this end, this work investigates the defect detection results of GKCPT when generating 20, 40, 60, 80, 100, 120, 140, 160 thermal images. It was found that the analysis results of the GKPCT method had the largest *SNR* values when 80 thermal images were generated. Some thermal images generated by SNGAN are shown in Figure 10. As with the original thermal image, the generated thermal image contains noise and severe background inhomogeneity, which makes it difficult to visually detect defective features in the image.

**Figure 11:**
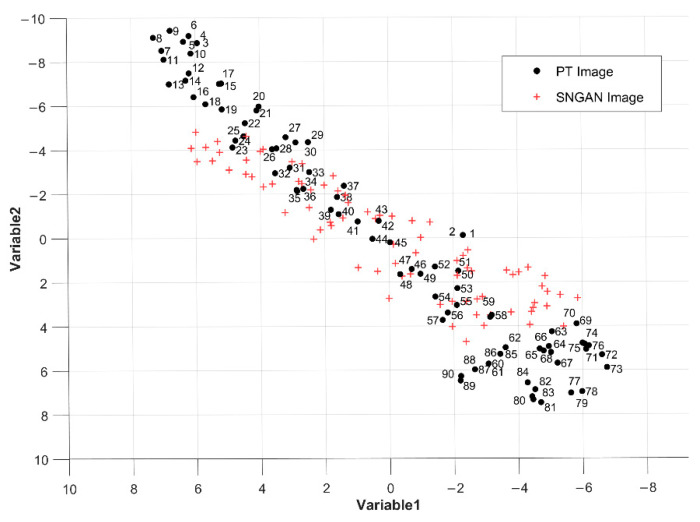
The t-SNE visualization of PT thermograms and SNGAN thermograms.

To further study the characteristics of SNGAN-generated data, t-distributed stochastic neighborhood embedding (t-SNE) is used to visualize the distribution of original and generated data [35]. t-SNE results for 2D visualization are shown in Figure 11, where the order of original thermograms in the time series is marked with numbers. The SNGAN-generated images in the figure are distributed around the middle of the original PT image sequence. A reasonable explanation is that the first few frames of the PT test thermograms contain a lot of noise and inhomogeneous background, while the last few frames have very little temperature decay. Compared to the two phases, the thermograms in the middle period contain more defect detection information and become the main information for SNGAN training and learning. Eventually, SNGAN generates thermal images with the same distribution of training data as the middle part.

**Figure 12:**
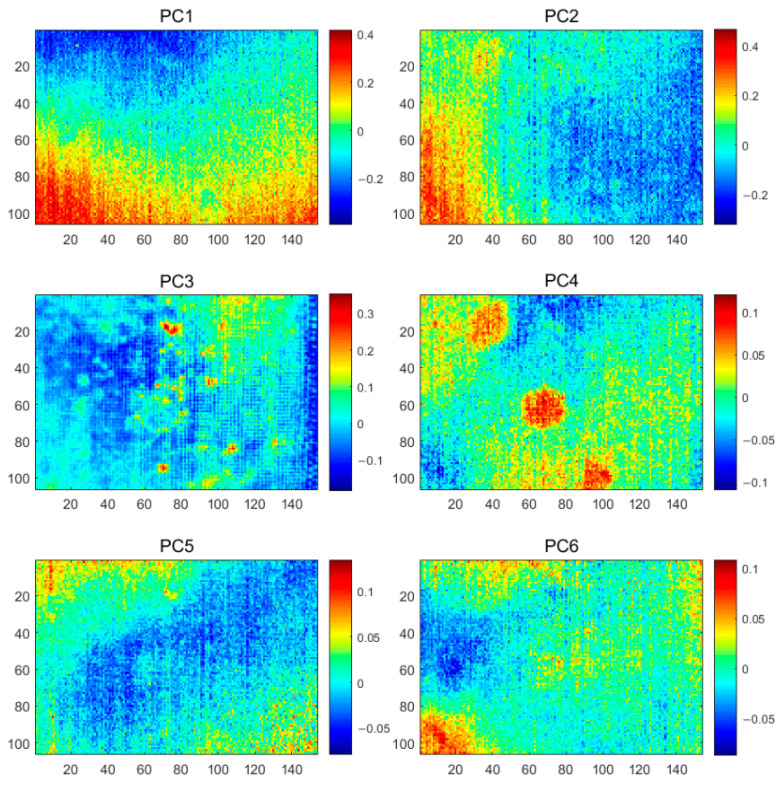
The GKPCT analysis results of the CFRP specimen 1.

The results of the proposed GKPCT method are shown in Figure 12, where the PC4 image extracts the morphology of all defects with clear defect boundaries. The PC1 and PC2 images have extracted uneven background. Compared to GPCT and KPCT, the inhomogeneous background information is reduced in the GKPCT results, which is attributed to the fact that SNGAN expands the dataset to increase the training data of the model and reduces the degree of information imbalance. Additionally, although this paper is to validate the feasibility of GKPCT by man-made defects, some defects not intended for man-made purposes can also be detected (cavity defects showed by PC3 in Figure 12). This also indirectly indicates the ability of GKPCT to be generalized in practical applications. These results show that GKPCT has a good ability to separate information and identify defects, which is an effective thermographic data analysis method that can improve IRT’s ability to evaluate internal defects in CFRP specimen.

Table 1 compares the *SNR* values of the original thermal images and several methods, and the largest *SNR* values obtained by each method are listed in the table. The *SNR* values and defect visualization map performance of each method are basically the same: 1. PCT detects the presence of defects compared to the original thermal image, and performs well especially in the detection of diamond-shaped defects (which is superior to other methods); 2. GPCT, a data-augmented PCT method, detects a large improvement in the contour and clarity of defects in the resulting thermal image compared to PCT (in the detection of circular defects good), but the *SNR* value is calculated to be small due to the severe inhomogeneous background in the thermal image masking the defects; 3. KPCT, a method proposed for the characteristics of the nonlinearity of thermal image data, the defect information is effectively separated from the inhomogeneous background, noise and other information. Additionally, the inspection results are concentrated in one thermal image, which saves inspection time and effort. However, subject to the factor of insufficient training data, the visibility of defects in the detection results of the KPCT method is still low, which in scenarios requiring higher detection accuracy, the accurate detection of defects can only depend on the knowledge and skills of the inspector and can easily lead to ambiguity in the defect evaluation results; 4. GKPCT, a data-augmented and nonlinear PCT method, has strong defect-recognition compared with several comparison methods capability (sub-high *SNR* for diamond-shaped defects, similar to the detection results of PCT methods). The method further improves the performance of the KPCT model in the task of defect detection and identification with the data augmentation strategy, which overcomes the challenge of insufficient training data and also solves the problem of difficult information separation due to the nonlinearity of thermal image data, and enhances the visibility of defects in the detection results.

### 4.2. Performance Analysis of GKPCT Method on Specimen 1

The GKPCT method solves the problem of insufficient model training data and performs well in the evaluation of nonlinear thermographic data defects. Generally, the increase in training data enables the model to better learn and fit data features. However, it is important to ensure that the additional training data contains valid information that can help improve model performance. For the task of IRT defect detection, the accurate identification of defects lies in comparing the local temperature information in the thermogram. In this work, a local pixel standard deviation (LPSD) index is introduced to measure the local smoothness of the thermal image.

**Figure 13:**
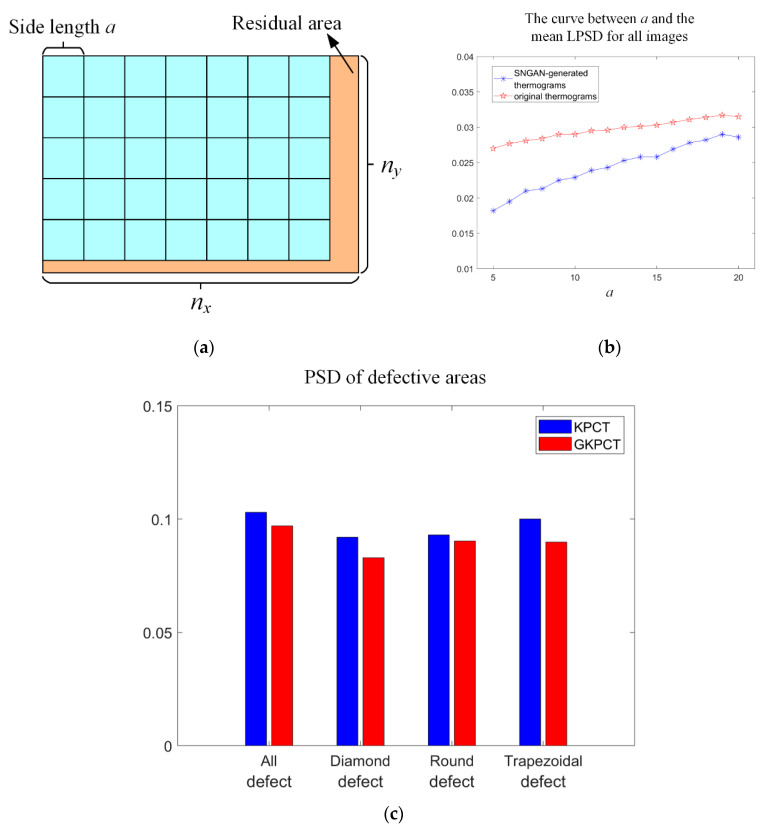
(**a**) Schematic diagram of area division; (**b**) the relationship between LPSD and *a*; (**c**) the PSD of the defective areas in KPCT and GKPCT.

The LPSD design process is exemplified in Figure 13a with the following details. First, the pixel values of the thermal images are Min-Max normalized to (0, 1). Each image is then divided into several square regions with side length *a*, and the pixel standard deviation (PSD) values in each region is calculated. Note that the value of a should not be set too large, otherwise LPSD loses its usefulness as a measure of local quality. Finally, the average value of all PSD is taken as the LSPD value of the image, which is a measure of the smoothness of the image. Thus, the LPSD is calculated as:

(9) $$\mathrm{LPSD}=\frac{\sum_{i=1}^{n}\sigma_{\mathrm{local}\text{\_}}{}_{i}}{n},$$

where *n* is the number of squares blocks with side length *a* obtained after partitioning and *n* = floor (*n_y_*/*a*), here, floor is a downward integer function that returns the largest integer not larger than the result, *n_y_* is the length of the thermogram. $\sigma_{\mathrm{local}\text{\_}i}$ is the standard deviation of the pixel values contained within the *i*-th square. A small LPSD value means that the image is smoother in the local area, or it can be understood that the pixel values of the local area are not discrete, and the concentrated pixel values are reflected in the thermogram as almost the same temperature color. The local difference helps to identify the contour of the defect.

As shown in Figure 13b, the LPSD values of the thermal images (i.e., 90 PT thermal images and 80 SNGAN thermal images) used for the GKPCT modeling were calculated and plotted in the figure, respectively. It can be seen that the LPSD values of the SNGAN-generated thermal images are much smaller when *a* is set from 5 to 30, which means that the SNGAN images have a locally higher smoothness compared to the original images. This may be due to the low noise of the generated thermal image caused by the convolution operation included in the SNGAN structure. To further investigate the promotion of SNGAN image augmentation on GKPCT, the PSD values of defective regions (local locations) in the PC4 images of KPCT and GKPCT were calculated separately. The comparison between the two is shown in Figure 13c, where the pixel standard deviation value of GKPCT is lower for both individual defects and the entire defect region. This indicates that the pixels in the defect area are numerically closer and the color of the defect area becomes more visually consistent. As a result, GKPCT has a better defect identification ability than KPCT, and the size and boundaries of defects are clearer in its visualization results.

### 4.3. Thermal Image Data Analysis of Specimen 2

**Figure 14:**
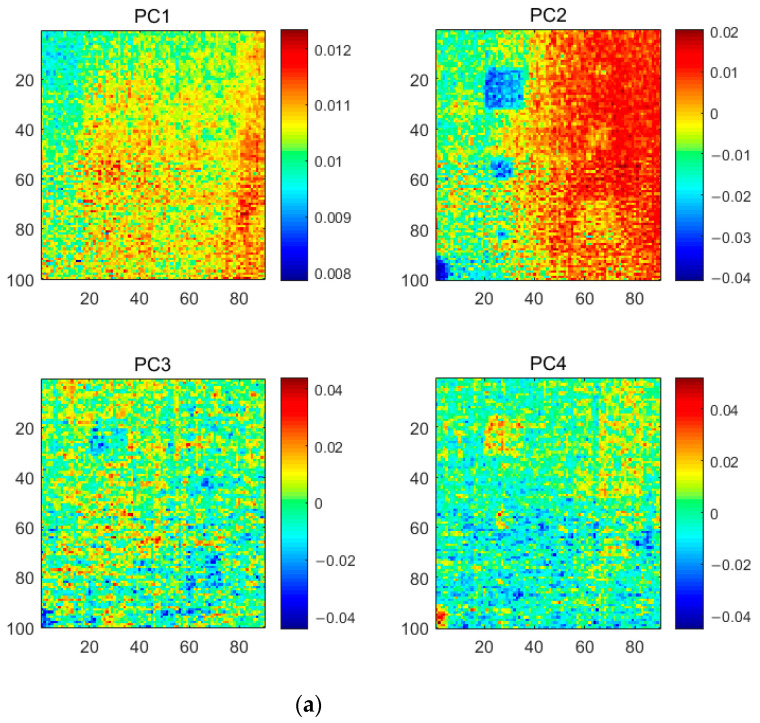

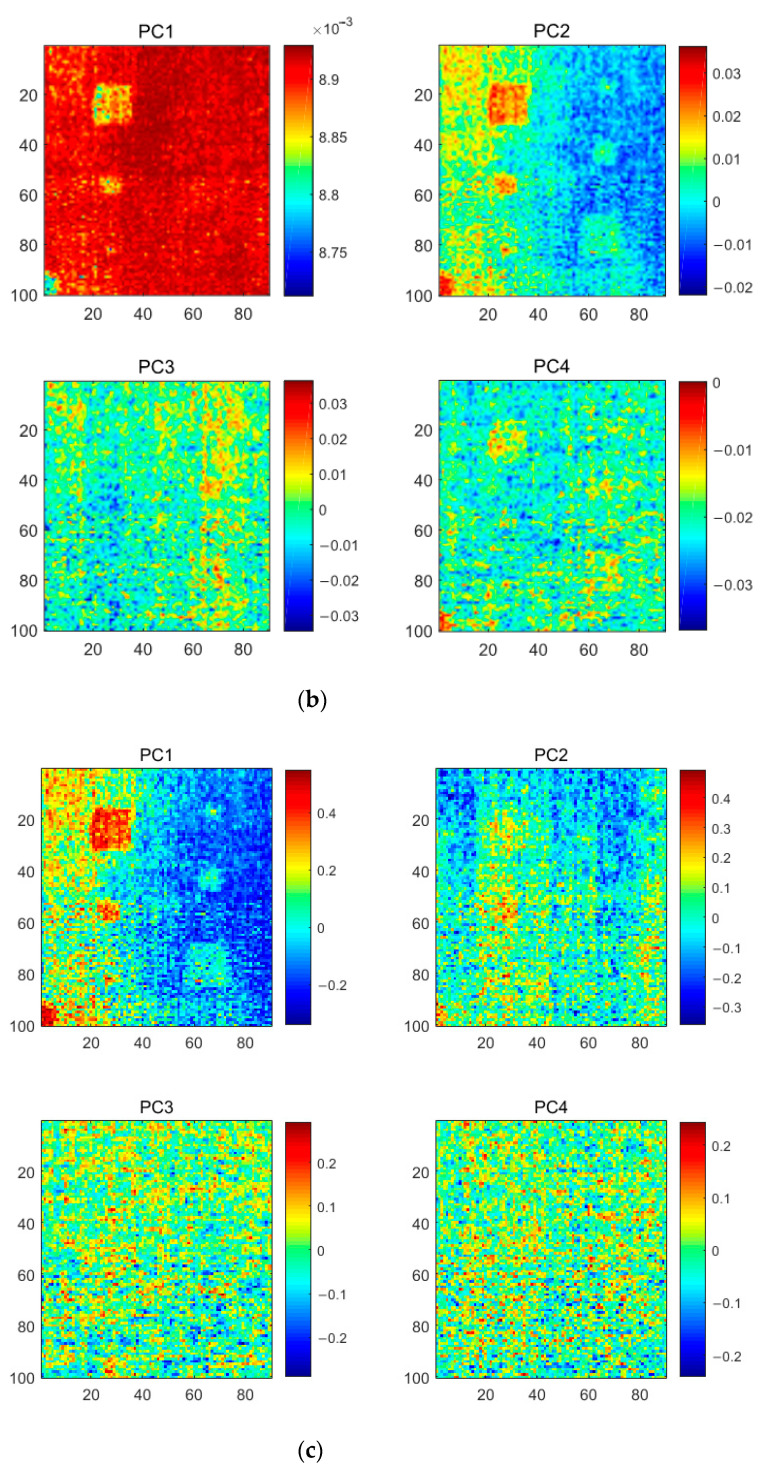

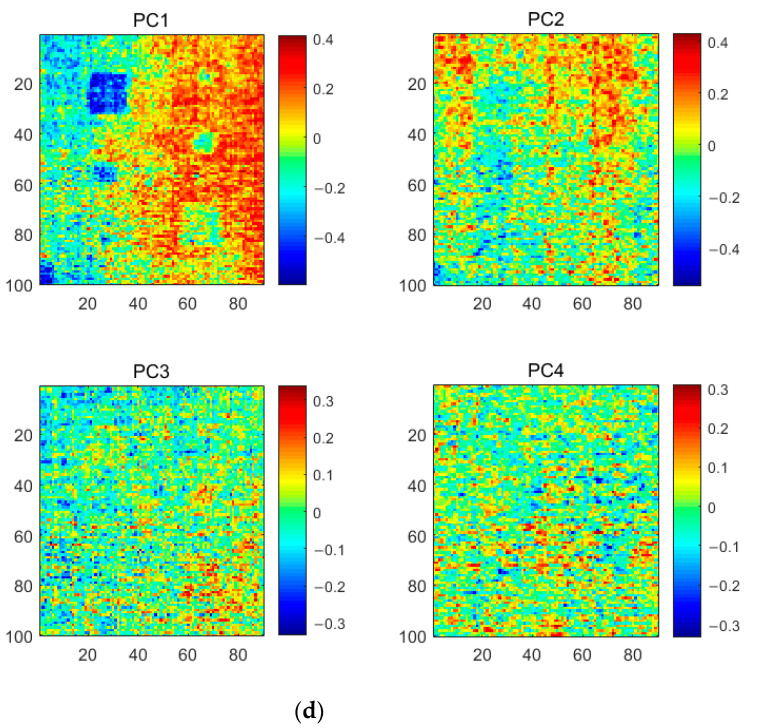
Analysis results of CFRP Specimen 2 based on (**a**) PCT, (**b**) GPCT, (**c**) KPCT (with a Gaussian kernel whose width is set to 1/τ=0.2), and (**d**) GKPCT (1/τ=0.2 ).

Similar to the analysis procedure of specimen 1, first, the GKPCT method was performed to analyze the PT results of CFRP specimen 2. In detail, SNGAN was used to generate 60 fake thermograms which were combined with the original thermograms. In doing this, the dataset was enlarged, which was then analyzed by implementing KPCT. The results are shown in Figure 14. The processing results of PCT, GPCT, and KPCT are depicted in the same figure for comparison. It is observed that the results of GKPCT have the best visibility of the defects, especially the three defects in the right side which locate deeper than those in the left. The quantitative evaluation results based on the *SNR* index are listed in Table 2. GKPCT has the largest *SNR* values for defects A2, B2, and C2. In the meantime, its overall performance is also the best. However, concerning defects A1, B1, and C1, the *SNR* values associated with GKPCT are not the largest, although these defects can be easily identified from the feature images provided by GKPCT as shown in Figure 14. The reason why the *SNR* values are against the intuition is attributed to the fact that *SNR* compares the defect area with the global background, whereas actually the human judgment of the defects only compares the defective regions with the neighboring area. This is a major disadvantage of the *SNR* index.

**Figure 15:**
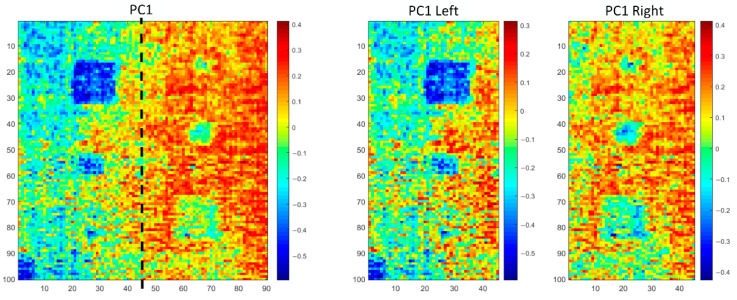
Schematic diagram of image segmentation with the improved *SNR* calculation.

The above discussions can be verified by equally dividing the image into left and right parts and calculating the *SNR* values for each method based on the half image, as illustrated in Figure 15. In other words, the *SNR* values associated with defects A1, B1, and C1 are calculated based on the left half image alone, while the *SNR* values of A2, B2, and C2 are calculated based on the right half image. The results are shown in Table 3, from which it is obvious that GKPCT outperforms other methods.

In contrast to the *SNR* index, the LPSD index proposed in this work focuses more on the contrast between the defect region and its surrounding neighbors, which is in line with the human intuition. The LPSD values of different methods are listed in Table 4, where the results show the outer performance of the proposed GKPCT method.

## 5. Conclusions

In this work, a novel nonlinear thermographic data analysis method, namely GKPCT, is proposed to improve the detection accuracy of subsurface defects in CFRP. The SNGAN-generated images are mixed with real thermal images for better feature extraction based on nonlinear dimensionality reduction, which results in enhanced defect detection performance. The experimental results of two CFRP experiments show that the combination of image augmentation and nonlinear analysis of thermal images helps to extract features and improve the visibility of defects in the detection results. In the future, the efficacy of the method on other types of composites will be further tested.

## Figures and Tables

**Table 1:** *SNR* values of different methods for CFRP specimen 1.

	*SNR*			
	Diamond Defect	Circular Defect	Trapezoidal Defect	All Defects
Original images	1.50	0.48	1.14	0.72
PCT [11]	1.97	2.04	3.35	1.67
GPCT [13]	1.74	2.56	2.25	1.35
KPCT	1.85	2.22	2.33	2.00
GKPCT	1.91	2.61	3.64	2.25

**Table 2:** *SNR* values of different methods for CFRP specimen 2.

	*SNR*						
	Defect A1	Defect B1	Defect C1	Defect A2	Defect B2	Defect C2	All Defects
Original images	0.83	0.93	1.09	0.84	0.90	1.06	0.17
PCT [11]	1.67	1.52	1.33	1.25	1.27	1.14	0.67
GPCT [13]	2.01	1.82	**1.57**	1.32	1.45	1.22	1.16
KPCT	**2.17**	**1.86**	1.46	1.40	1.53	1.19	1.22
GKPCT	1.94	1.78	1.54	**1.43**	**1.56**	**1.21**	**1.27**

The value in bold indicates the maximum *SNR* value corresponding to the defect.

**Table 3:** *SNR* values based on half images.

	*SNR*						
	Defect A1	Defect B1	Defect C1	Defect A2	Defect B2	Defect C2	All Defects
PCT [11]	1.83	1.74	1.49	1.18	1.02	0.96	0.67
GPCT [13]	2.08	1.92	1.68	1.24	1.29	1.07	1.16
KPCT	2.25	1.99	1.57	1.35	1.47	1.13	1.22
GKPCT	**2.54**	**2.18**	**1.71**	**1.46**	**1.60**	**1.28**	**1.27**

The value in bold indicates the maximum *SNR* value corresponding to the defect.

**Table 4:** LPSD values of different methods for CFRP specimen 2.

	LPSD						
	Defect A1	Defect B1	Defect C1	Defect A2	Defect B2	Defect C2	All Defects
PCT [11]	0.0112	0.0131	0.0111	0.0122	0.0123	0.0123	0.0114
KPCT	0.1390	0.1716	0.1422	0.1011	0.0949	0.0891	0.1772
GKPCT	**0.1561**	**0.2081**	**0.1731**	**0.1279**	**0.1324**	**0.1035**	**0.1973**

The value in bold indicates the maximum LPSD value corresponding to the defect.

## Data Availability

The data presented in this study are available on request from the corresponding author.
